# Swimtrans Net: a multimodal robotic system for swimming action recognition driven via Swin-Transformer

**DOI:** 10.3389/fnbot.2024.1452019

**Published:** 2024-09-24

**Authors:** He Chen, Xiaoyu Yue

**Affiliations:** ^1^Department of Physical Education, Sangmyung University, Seoul, Republic of Korea; ^2^Nanjing University of Technology, Nanjing, Jiangsu, China

**Keywords:** Swin-Transformer, CLIP, multimodal robotic, swimming action recognition, transfer learning

## Abstract

**Introduction:**

Currently, using machine learning methods for precise analysis and improvement of swimming techniques holds significant research value and application prospects. The existing machine learning methods have improved the accuracy of action recognition to some extent. However, they still face several challenges such as insufficient data feature extraction, limited model generalization ability, and poor real-time performance.

**Methods:**

To address these issues, this paper proposes an innovative approach called Swimtrans Net: A multimodal robotic system for swimming action recognition driven via Swin-Transformer. By leveraging the powerful visual data feature extraction capabilities of Swin-Transformer, Swimtrans Net effectively extracts swimming image information. Additionally, to meet the requirements of multimodal tasks, we integrate the CLIP model into the system. Swin-Transformer serves as the image encoder for CLIP, and through fine-tuning the CLIP model, it becomes capable of understanding and interpreting swimming action data, learning relevant features and patterns associated with swimming. Finally, we introduce transfer learning for pre-training to reduce training time and lower computational resources, thereby providing real-time feedback to swimmers.

**Results and discussion:**

Experimental results show that Swimtrans Net has achieved a 2.94% improvement over the current state-of-the-art methods in swimming motion analysis and prediction, making significant progress. This study introduces an innovative machine learning method that can help coaches and swimmers better understand and improve swimming techniques, ultimately improving swimming performance.

## 1 Introduction

Swim motion recognition, as an important research field in motion pattern analysis, holds both academic research value and practical application demand. Swimming is a widely popular sport worldwide (Valdastri et al., 2011). However, in practical training and competitions, capturing and evaluating the technical details of swim motions accurately can be challenging (Colgate and Lynch, 2004). Therefore, utilizing advanced motion recognition techniques for swim motion analysis can not only help athletes optimize training effectiveness and improve performance but also provide scientific evidence in sports medicine to effectively prevent sports injuries. Additionally, swim motion recognition technology can assist referees in making fair and accurate judgments during competitions (Chowdhury and Panda, 2015). Thus, research and development in swim motion recognition not only contribute to the advancement of sports science but also bring new opportunities and challenges to the sports industry.

The initial methods primarily involved swim motion recognition through the use of symbolic AI and knowledge representation. Expert systems, which encode domain experts' knowledge and rules for reasoning and decision-making, are widely used symbolic AI approaches. For example, Feijen et al. (2020) developed an algorithm for online monitoring of swimming training that accurately detects swimming strokes, turns, and different swimming styles. Nakashima et al. (2010) developed a swim motion display system using wrist-worn accelerometer and gyroscope sensors for athlete training. Simulation-based approaches are also effective, as they involve building physical or mathematical models to simulate swim motions for analysis and prediction. Xu (2020) utilized computer simulation techniques, employing ARMA models and Lagrangian dynamics models, to analyze the kinematics of limb movements in swimming and establish a feature model for swim motion analysis. Jie (2016) created a motion model for competitive swim techniques using virtual reality technology and motion sensing devices, enabling swim motion simulation and the development of new swimming modes. Another approach is logistic regression, a statistical method used to analyze the relationship between feature variables and outcomes of swim motions by constructing regression models. Hamidi Rad et al. (2021) employed a single IMU device and logistic regression to estimate performance-related target metrics in various swimming stages, achieving high R^2^ values and low relative root mean square errors. While these techniques have the benefits of being methodical and easily understandable, they also come with the limitations of needing extensive background knowledge and complex computational requirements.

To address the drawbacks of requiring substantial prior knowledge and high computational complexity in the initial algorithms, data-driven and machine learning-based approaches in swim motion recognition primarily rely on training models with large amounts of data to identify and classify swim motions. These methods offer advantages such as higher generalization capability and automated processing. Decision tree-based methods perform motion recognition by constructing hierarchical decision rules. For example, Fani et al. (2018) achieved a 67% accuracy in classifying freestyle stroke postures using a decision tree classifier. Random forest-based methods enhance recognition accuracy by ensembling multiple decision trees. For instance, Fang et al. (2021) achieved high-precision motion state recognition with an accuracy of 97.26% using a random forest model optimized with Bayesian optimization. Multi-layer perceptron (MLP), as a type of feedforward neural network, performs complex pattern recognition through multiple layers of nonlinear transformations. Na et al. (2011) combined a multi-layer perceptron with a gyroscope sensor to achieve swim motion recognition for target tracking in robotic fish. Nevertheless, these approaches are constrained by their reliance on extensive annotated data, extended model training periods, and possible computational inefficiencies when handling real-time data.

To address the drawbacks of high prior knowledge requirements and computational complexity in statistical and machine learning-based algorithms, deep learning-based algorithms in swim motion recognition primarily utilize techniques such as Convolutional Neural Networks (CNN), reinforcement learning, and Transformers to automatically extract and process complex data features. This approach offers higher accuracy and automation levels. CNN extracts spatial features through deep convolutional layers. For example, Guo and Fan (2022) achieved a classification accuracy of up to 97.48% in swim posture recognition using a hybrid neural network algorithm. Reinforcement learning identifies swim motions by learning effective propulsion strategies. For instance, Gazzola et al. (2014) combined reinforcement learning algorithms with numerical methods to achieve efficient motion control for self-propelled swimmers. Rodwell and Tallapragada (2023) demonstrated the practicality of reinforcement learning in controlling fish-like swimming robots by training speed and path control strategies using physics-informed reinforcement learning. Transformers, with their powerful sequential modeling capability, can effectively process and recognize complex time series data. Alternative approaches have also been explored to overcome the limitations of deep learning models. For example, hybrid models that integrate classical machine learning techniques with deep learning frameworks have been proposed. Athavale et al. (2021) introduced a hybrid system combining Support Vector Machines (SVM) with CNNs to leverage the strengths of both methods, achieving higher robustness in varying swimming conditions. Additionally, edge computing and federated learning have been investigated to address the high computational resource demands, enabling more efficient real-time processing and preserving data privacy (Arikumar et al., 2022). Nevertheless, these techniques come with certain drawbacks such as their heavy reliance on extensive annotated datasets, demanding computational resources, and possible delays in response time for real-time tasks.

To address the issues of high dependency on large labeled datasets, high computational resource requirements, and insufficient response speed in real-time applications, we propose our method: Swimtrans Net - a multimodal robotic system for swimming action recognition driven by Swin-Transformer. By leveraging the powerful visual data feature extraction capabilities of Swin-Transformer, Swimtrans Net effectively extracts swimming image information. Additionally, to meet the requirements of multimodal tasks, we integrate the CLIP model into the system. Swin-Transformer serves as the image encoder for CLIP, and through fine-tuning the CLIP model, it becomes capable of understanding and interpreting swimming action data, learning relevant features and patterns associated with swimming. Finally, we introduce transfer learning for pre-training to reduce training time and lower computational resources, thereby providing real-time feedback to swimmers.

Contributions of this paper:

- Swimtrans Net innovatively integrates Swin-Transformer and CLIP model, offering advanced feature extraction and multimodal data interpretation capabilities for swimming action recognition.
- The approach excels in multi-scenario adaptability, high efficiency, and broad applicability by combining visual data encoding with multimodal learning and transfer learning techniques.
- Experimental results demonstrate that Swimtrans Net significantly improves accuracy and responsiveness in real-time swimming action recognition, providing reliable and immediate feedback to swimmers.

## 2 Related work

### 2.1 Action recognition

In modern sports, accurately analyzing and recognizing various postures and actions have become essential for enhancing athlete performance and training efficiency. Deep learning and machine learning models play a crucial role in this process (Hu et al., 2016). Specifically, in swimming, these technologies have made significant advancements. They effectively identify and classify different swimming styles such as freestyle, breaststroke, and backstroke, as well as specific movements like leg kicks and arm strokes. This detailed classification and recognition capability provide valuable training data and feedback for coaches and athletes (Dong et al., 2024). Studying feature extraction and pattern recognition methods for postures and actions is key to improving the accuracy and effectiveness of swimming motion analysis and prediction. Deep learning models can capture subtle motion changes and features by analyzing extensive swimming video data, enabling them to identify different swimming techniques. This helps coaches develop more scientific training plans and provides athletes with real-time feedback and correction suggestions (Wang et al., 2024). Moreover, advancements in wearable devices and sensor technology have made obtaining high-quality motion data easier. These devices can record specific actions and postures, providing rich training data for deep learning models. For instance, high-precision accelerometers and gyroscopes can record athletes' movements in real time, which are then analyzed by deep learning models.

### 2.2 Transformer models

Transformer models have revolutionized artificial intelligence, demonstrating exceptional performance and versatility across various domains. In natural language processing (NLP), they significantly enhance machine translation, text summarization, question answering, sentiment analysis, and language generation, leading to more accurate and context-aware systems (Hu et al., 2021). In computer vision, Vision Transformers (ViTs) excel in image recognition, object detection, image generation, and image segmentation, achieving state-of-the-art results and advancing fields like medical imaging and autonomous driving. For audio processing, transformers improve speech recognition, music generation, and speech synthesis, contributing to better virtual assistants and transcription services (Lu et al., 2024). In healthcare, transformers assist in medical image analysis, drug discovery, and clinical data analysis, offering precise disease detection and personalized medicine insights. The finance sector benefits from transformers through algorithmic trading, fraud detection, and risk management, enhancing security and decision-making. In gaming and entertainment, transformers generate storylines, dialogues, and level designs, enriching video games and virtual reality experiences. Lastly, in robotics, transformers enable autonomous navigation and human-robot interaction, advancing technologies in autonomous vehicles and drone navigation. Overall, the versatility and power of transformer models drive innovation and efficiency across a multitude of applications, making them indispensable in modern technology (Li et al., 2014).

### 2.3 Multimodal data fusion

Multimodal Data Fusion focuses on enhancing the analysis and prediction of swimming motions by utilizing data from various sources, such as images, videos, and sensor data (Hu et al., 2018). By integrating data from different modalities, researchers can obtain a more comprehensive and accurate understanding of swimming motions. For instance, combining images with sensor data allows for the simultaneous capture of a swimmer's posture and motion trajectory, leading to more thorough analysis and evaluation (Zheng et al., 2022). This approach can provide detailed insights into the efficiency and technique of the swimmer, which are crucial for performance improvement and injury prevention. Moreover, multimodal data fusion can significantly broaden the scope and capabilities of swimming motion analysis and prediction. It enables the development of advanced models that can interpret complex motion patterns and provide real-time feedback to swimmers and coaches. This, in turn, facilitates the creation of personalized training programs tailored to the individual needs of each swimmer, enhancing their overall performance. Research in this area continues to push the boundaries of what is possible in sports science, promising more sophisticated tools for analyzing and optimizing athletic performance (Nguyen et al., 2016). Overall, the integration of multimodal data represents a significant advancement in the field, offering a richer, more nuanced understanding of swimming motions and contributing to the advancement of sports technology and training methodologies.

## 3 Methodology

### 3.1 Overview of our network

This study proposes a deep learning-based method, Swimtrans Net: a multimodal robotic system for swimming action recognition driven via Swin-Transformer, for analyzing and predicting swimming motions. This method combines the Swin-Transformer and CLIP models, leveraging their advantages in image segmentation, feature extraction, and semantic understanding to provide a more comprehensive and accurate analysis and prediction of swimming motions. Specifically, the Swin-Transformer is used to extract and represent features from swimming motion data, capturing the spatial characteristics of the actions. Then, the CLIP model is introduced to understand and interpret the visual information in the swimming motion data, extracting the semantic features and techniques of the actions. Finally, transfer learning is used to apply the pre-trained Swin-Transformer and CLIP models to the swimming motion data, and model parameters are fine-tuned to adapt them to the specific tasks and data of swimming motions.

First of all, Collect datasets containing swimming motions in the form of videos, sensor data, etc., and preprocess the data by removing noise, cropping, and annotating action boundaries to prepare it for model training and testing. Use the Swin-Transformer model to extract and represent features from the swimming motion data, decomposing it into small patches and capturing relational information through a self-attention mechanism to effectively extract spatial features. Introduce the CLIP model and input the swimming motion data into it; by learning the correspondence between images and text, the CLIP model can perform semantic understanding and reasoning of the image data. Applying the CLIP model to the swimming motion data helps the system better understand the action features and techniques in swimming motions. Apply the pre-trained Swin-Transformer and CLIP models to the swimming motion data, and use transfer learning and fine-tuning to adapt them to the specific tasks and data of swimming motions, improving the model's performance in analysis and prediction. Finally, evaluate the trained model by comparing it with actual swimming motions, assessing its performance in analysis and prediction tasks, and apply this method to actual swimmers and coaches, providing accurate technique evaluations and improvement suggestions.

The term “robotic system” was chosen to emphasize the integration of advanced machine learning models with automated hardware components, creating a cohesive system capable of autonomous analysis and prediction of swimming motion data. Our system leverages both the Swin-transformer and CLIP models to process and interpret the data, which is then used by the robotic components to provide real-time feedback and analysis to swimmers. By referring to it as a “robotic system,” we aim to highlight the seamless collaboration between software algorithms and physical devices (such as cameras, sensors, and possibly robotic feedback mechanisms) that together perform complex tasks with minimal human intervention. This terminology helps to convey the sophisticated and automated nature of the system, distinguishing it from purely software-based solutions.

### 3.2 Swin-Transformer model

**Figure 1:**
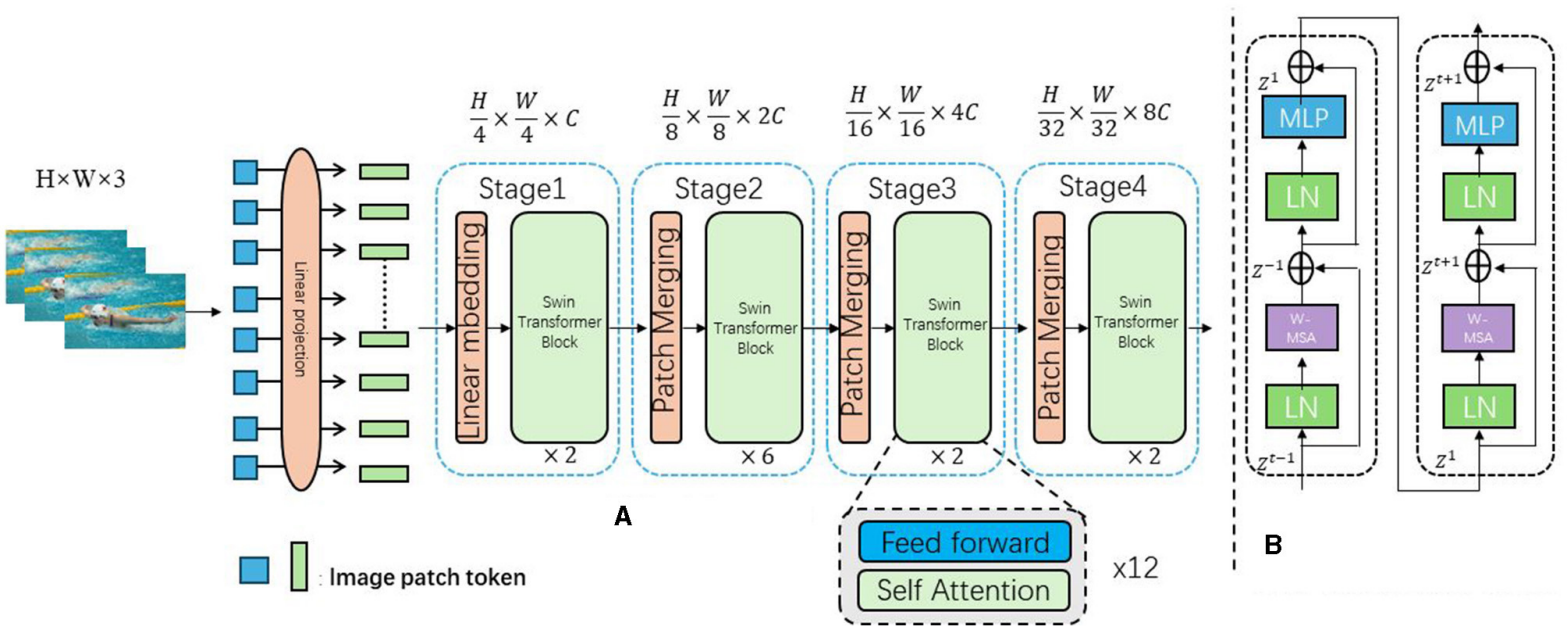
The swimming action image is input, segmented into small blocks by Swin-Transformer, and the self-attention mechanism is applied to extract features, which are then used for action understanding, semantic extraction and prediction. **(A)** Architecture. **(B)** Two successive Swin-Transformer blocks.

Swin-Transformer (Swin Attention Mechanism) is an image segmentation and feature extraction model based on self-attention mechanisms, playing a crucial role in swimming motion analysis and prediction methods (Tsai et al., 2023). Figure 1 is a schematic diagram of the principle of Swin-Transformer Model.

**Figure 2:**
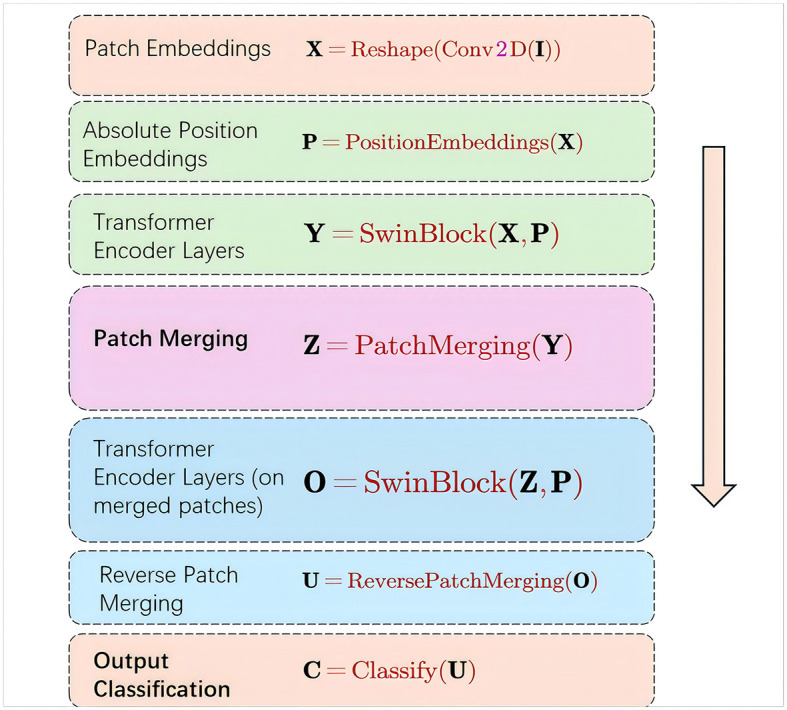
Schematic diagram of the calculation process of Formula 1-7.

The Swin-Transformer leverages self-attention mechanisms to capture the relational information between different regions of an image, enabling image segmentation and feature extraction. Unlike traditional convolutional neural networks (CNNs) that rely on fixed-size convolution kernels, the Swin-Transformer divides the image into a series of small patches and establishes self-attention connections between these patches. The core idea of the Swin-Transformer is to establish a global perception through a multi-level attention mechanism. Specifically, it uses two types of attention mechanisms: local attention and global attention. Local attention captures the relational information within patches, while global attention captures the relational information between patches. This multi-level attention mechanism allows the Swin-Transformer to understand the semantics and structure of images from multiple scales. In the context of swimming motion analysis and prediction, the Swin-Transformer model plays a crucial role in extracting and representing features from swimming motion data. By decomposing the swimming motion data into small patches and applying the self-attention mechanism, the Swin-Transformer captures the relational information between different parts of the swimming motion and extracts spatial features of the motion. These features are then used for subsequent tasks such as motion understanding, semantic extraction, and prediction, enabling accurate analysis and prediction of swimming motions (Figure 2).


(1) $$\begin{array}{l} \text{Patch Embeddings}:\text{X}=\text{Reshape}\left(\text{Conv2D}\left(\text{I}\right)\right) \end{array}$$

The patch embeddings operation takes an input image **I** and applies a convolutional operation to extract local features. The resulting feature map is then reshaped to obtain a sequence of patch embeddings **X**.


(2) $$\begin{array}{l} \text{Absolute Position Embeddings}:\text{P}=\text{PositionEmbeddings}\left(\text{X}\right) \end{array}$$

The absolute position embeddings operation generates a set of learnable position embeddings **P** that encode the absolute position information of each patch in the sequence.


(3) $$\begin{array}{l} \text{transformerer Encoder Layers}:\text{Y}=\text{SwinBlock}\left(\text{X},\text{ P}\right) \end{array}$$

The Swin-Transformerer encoder layers, implemented as SwinBlocks, take the patch embeddings **X** and absolute position embeddings **P** as inputs. These layers apply self-attention and feed-forward neural networks to enhance the local and global interactions between patches, resulting in the transformered feature representations **Y**.


(4) $$\begin{array}{l} \text{Patch Merging}:\text{Z}=\text{PatchMerging}\left(\text{Y}\right) \end{array}$$

The patch merging operation combines neighboring patches in the transformered feature map **Y** to obtain a lower-resolution feature map **Z**. This helps capture long-range dependencies and reduces computational complexity.


(5) $$\begin{array}{c} \text{transformerer Encoder Layers (on merged patches)}:\text{O}=\\ \text{SwinBlock}\left(\text{Z},\text{ P}\right) \end{array}$$

The Swin-Transformerer encoder layers are applied again, but this time on the merged patch embeddings **Z** using the same absolute position embeddings **P**. This allows for further refinement of the feature representations, considering the interactions between the merged patches.


(6) $$\begin{array}{l} \text{Reverse Patch Merging}:\text{U}=\text{ReversePatchMerging}\left(\text{O}\right) \end{array}$$

The reverse patch merging operation restores the feature map resolution by reversing the patch merging process, resulting in the refined high-resolution feature map **U**.


(7) $$\begin{array}{l} \text{Output Classification}:\text{C}=\text{Classify}\left(\text{U}\right) \end{array}$$

Finally, the high-resolution feature map **U** is fed into a classification layer to obtain the output classification probabilities **C**.

By introducing the Swin-Transformer model, the swimming motion analysis method can better utilize the spatial information of image data, extracting richer and more accurate feature representations. This helps to improve the performance of swimming motion analysis and prediction, providing swimmers and coaches with more accurate technical evaluations and improvement guidance.

### 3.3 CLIP

**Figure 3:**
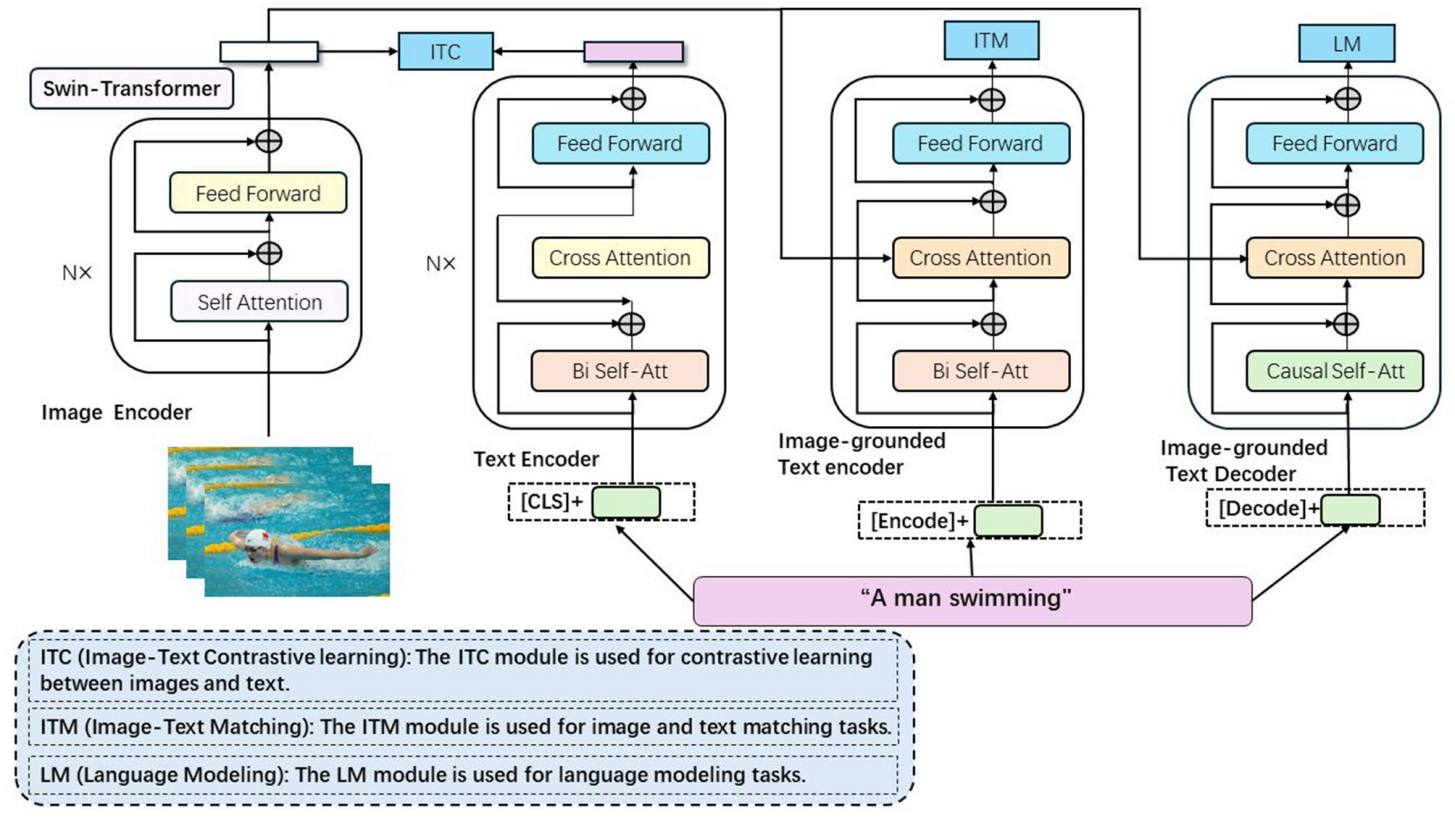
The image is encoded into a vector through Swin Transformer, and the text is converted into a vector through the text encoder. After being fused through the ITC, ITM, and LM modules, the alignment and generation of the image and text are achieved.

CLIP (Contrastive Language-Image Pretraining) (Kim et al., 2024a) is a model designed for image and text understanding based on contrastive learning, playing a critical role in swimming motion analysis and prediction methods (shown in Figure 3). The model achieves cross-modal semantic understanding and reasoning by learning the correspondence between images and text through a unified embedding space. This capability allows the model to effectively interpret and predict swimming motions by leveraging both visual and textual information, enhancing the accuracy and robustness of the analysis.

This space allows for measuring the similarity between images and text, enabling a combined representation of visual and semantic information. The image encoder utilizes a Swin Transformer to convert input images into vector representations, extracting features through several layers of self-attention and feed-forward operations, and mapping these features into vector representations in the embedding space. The text encoder processes input text into vector representations using self-attention mechanisms and feed-forward networks to model semantic relationships within the text. The Image-Text Contrastive (ITC) module aligns the image and text representations within the embedding space, ensuring that corresponding image-text pairs are closely positioned while non-matching pairs are far apart. The Image-Text Matching (ITM) module fine-tunes this alignment by incorporating cross-attention mechanisms, enhancing the model's ability to match images with their corresponding textual descriptions. The Language Modeling (LM) module uses image-grounded text encoding and decoding mechanisms, leveraging cross-attention and causal self-attention to generate text based on the given image, thereby enhancing the model's language generation capabilities with visual context. In the swimming motion analysis and prediction method, the model interprets visual information from swimming motion data by converting these visual features into vector representations within the embedding space. Textual descriptions of swimming techniques are similarly processed by the text encoder. This unified representation of visual and semantic information facilitates the analysis and prediction of swimming motions. By comparing the vector representation of a swimmer's actions with those of standard techniques or known movements, the model can assess the swimmer's technical level and provide suggestions for improvement. This is achieved by measuring the similarity between image and text vectors in the embedding space, enabling semantic understanding and reasoning of swimming actions.

ITC (Image-Text Contrastive Learning): The ITC module is used for contrastive learning between images and text. By comparing the output features of the image encoder and the text encoder, this module is able to align images and text in the embedding space, thereby achieving cross-modal contrastive learning. ITM (Image-Text Matching): The ITM module is used for image and text matching tasks. This module fuses image and text features through bi-directional self-attention (Bi Self-Att) and cross-attention (Cross Attention) mechanisms to determine whether the image and text match, thereby enhancing the model's cross-modal understanding ability. LM (Language Modeling): The LM module is used for language modeling tasks. This module generates text descriptions based on the contextual information provided by the image encoder through the causal self-attention (Causal Self-Att) mechanism, enhancing the model's text generation ability. Each module in the diagram consists of self-attention and feed-forward neural networks (Feed Forward), and implements specific functions through different attention mechanisms (such as cross-attention and bi-directional self-attention). These modules work together to complete the joint modeling of images and texts, improving the performance of the model in swimming motion analysis and prediction tasks.


(8) $$\begin{array}{l} \text{Image Encoder}:\text{v}={\text{Encoder}}_{\text{image}}\left(\text{I}\right) \end{array}$$

The image encoder operation takes an input image **I** and applies an encoder function Encoder_image_ to obtain the corresponding image embedding vector **v**.


(9) $$\begin{array}{l} \text{Text Encoder}:\text{t}={\text{Encoder}}_{\text{text}}\left(\text{text}\right) \end{array}$$

The text encoder operation takes an input text text and applies an encoder function Encoder_text_ to obtain the corresponding text embedding vector **t**.


(10) $$\begin{array}{l} \text{Similarity Score}:\text{score}=\text{CosineSimilarity}\left(\text{v},\text{ t}\right) \end{array}$$

The similarity score operation calculates the cosine similarity between the image embedding vector **v** and the text embedding vector **t**. This score represents the similarity or compatibility between the image and the text.


(11) $$\begin{array}{l} \text{Optimization Objective}:L=-\text{log}\left(\text{score}\right) \end{array}$$

The optimization objective is defined as the negative logarithm of the similarity score. The goal is to maximize the similarity score, which corresponds to minimizing the loss L.

CLIP leverages this framework to enable cross-modal understanding and reasoning between images and text, making it a powerful tool for tasks such as image-text retrieval, image classification based on textual descriptions, and more. By incorporating the CLIP model, the swimming motion analysis method can better utilize the semantic relationships between image and text data, extracting richer and more accurate action features. This helps to improve the performance of swimming motion analysis and prediction, providing swimmers and coaches with more accurate technical evaluations and improvement guidance.

### 3.4 Transfer learning

Transfer learning (Manjunatha et al., 2022) is a machine learning method that involves applying a model trained on a large-scale dataset to a new task or domain. The fundamental principle of transfer learning is to utilize the knowledge already learned by a model (Zhu et al., 2021), transferring the experience gained from training on one task to another related task. This accelerates the learning process and improves performance on the new task.

**Figure 4:**
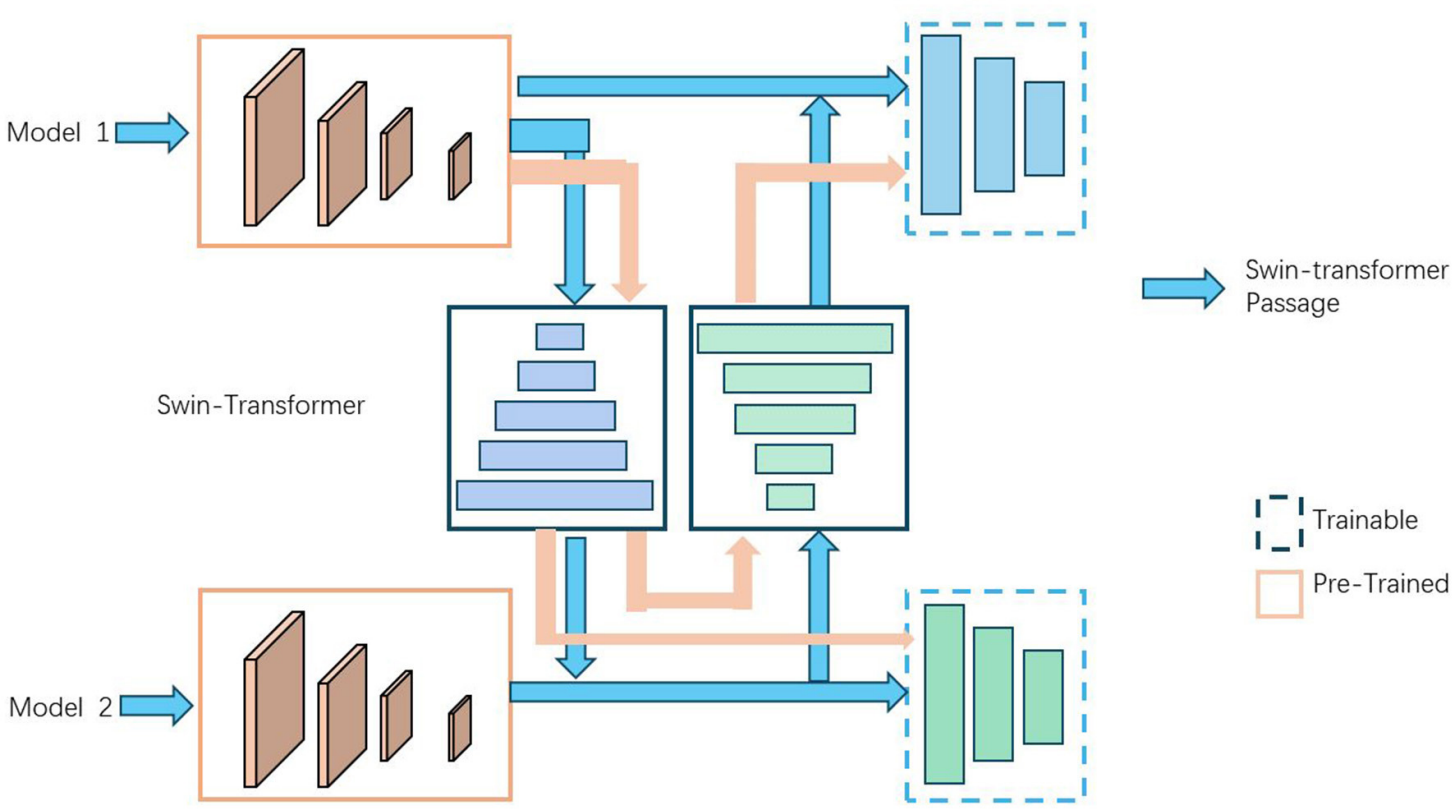
A schematic diagram of the principle of Transfer Learning.

Figure 4 is a schematic diagram of the principle of Transfer Learning.

In traditional machine learning, training a model requires a large amount of labeled data and computational resources. However, obtaining large-scale labeled data and training a complex model is often very expensive and time-consuming. This is why transfer learning has become highly attractive. By using a pre-trained model, we can leverage the parameters learned from existing data and computational resources, thereby quickly building and optimizing models for new tasks with relatively less labeled data and computational resources. The method illustrated in the image applies transfer learning to provide initial model parameters or assist in training the new task by transferring already learned feature representations and knowledge. There are several ways this can be done: using a pre-trained model as a feature extractor, where the initial layers learn general feature representations and the later layers are fine-tuned; fine-tuning the entire pre-trained model to optimize it on the new task's dataset; and domain adaptation, which adjusts the model's feature representation to better fit the new task's data distribution. The diagram demonstrates the use of a Swin-Transformer in conjunction with two models, highlighting the flow of data and the stages where transfer learning is applied. The Swin-Transformer acts as a central component, facilitating the transfer of learned features and knowledge between the pre-trained and trainable components of the models, ultimately optimizing performance for new tasks.


(12) $$\begin{array}{l} \theta^{\prime }=\arg\underset{\theta^{\prime }}{\min}L\left(\theta^{\prime },D_{\text{target}}\right) \end{array}$$

In this formula, θ′ represents the model parameters of the new task, L represents the loss function, and *D*_target_ represents the dataset of the new task.


(13) $$\begin{array}{l} \theta^{\prime }=\arg\underset{\theta^{\prime }}{\min}\left[\lambda L\text{source}\left(\theta^{\prime },D\text{source}\right)+\left(1-\lambda \right)L\text{target}\left(\theta^{\prime },D\text{target}\right)\right] \end{array}$$

This formula is the transfer learning formula when training with the source domain dataset (*D*_source_) and the target domain dataset (*D*_target_). λ is a hyperparameter that weighs the loss of the source domain and the target domain. Lsource and Ltarget represent the loss functions of the source domain and the target domain, respectively.

In Equation 11, the optimization objective is defined as the negative logarithm of the similarity score. The goal is to maximize the similarity score, which corresponds to minimizing the loss L. Here L is a general loss function used to maximize the similarity score. This loss function is implemented by minimizing the negative logarithm of the similarity score. In Equation 13, represents the loss function on the source data and target data, which are used for optimization of the source domain and target domain, respectively. Therefore, L appears repeatedly in these two places to describe the loss function in different contexts: one is a general similarity score loss, and the other is a specific application loss for the source data and target data.


(14) $$\begin{array}{c} \theta^{\prime }=\text{arg }{\text{min}}_{\theta^{\prime }}[\lambda ℒ\text{pretrain}(\theta^{\prime },D\text{pretrain})+\\ \left(1-\lambda )ℒ\text{target}(\theta^{\prime },D\text{target}\right)] \end{array}$$

This formula is the transfer learning formula when training with pre-trained model parameters (*D*_pretrain_) and target domain dataset (*D*_target_). $L_{\text{pretrain}}$ represents the loss function of the pre-trained model.

In these formulas, argmin represents the model parameter θ′ that minimizes the loss function. By minimizing the loss function, we can optimize the model parameters of the new task to better fit the data distribution of the target domain.

## 4 Experiment

### 4.1 Datasets

This article uses four datasets (Table 1): PKU-MMD Datasets, Sports-1M Dataset, UCF101 Dataset and Finegym Dataset. KU-MMD Dataset: (Liu et al., 2017) Description: PKU-MMD is a large-scale dataset for continuous multi-modality 3D human action understanding. It contains over 1,000 action sequences and covers a wide range of actions performed by different subjects. Usage: This dataset can be used to pre-train models on a variety of human motions, providing a robust foundation for understanding and recognizing complex swimming actions. Sports-1M Dataset: (Li et al., 2021) Description: Sports-1M is a large-scale video dataset with over one million YouTube sports videos categorized into 487 sports labels. It provides a diverse set of sports-related video clips. Usage: The Sports-1M dataset can be utilized for initial training of video recognition models, leveraging the vast diversity of sports actions to enhance the model's generalization capabilities for swimming motion analysis. UCF101 Dataset: (Safaei et al., 2020) Description: UCF101 is an action recognition dataset of realistic action videos collected from YouTube, containing 101 action categories. It is widely used for action recognition tasks. Usage: This dataset can be used to fine-tune models on action recognition tasks, specifically targeting the accurate recognition and classification of swimming strokes and techniques. Finegym Dataset: (Shao et al., 2020) Description: Finegym is a fine-grained action recognition dataset for gymnastic actions. It focuses on high-quality annotated videos of gymnastic routines. Usage: Finegym can be used to further fine-tune models to recognize and differentiate subtle differences in motion techniques, which is critical for detailed swimming motion analysis.

**Table 1:** Description and usage of datasets.

**Dataset**	**Description**	**Usage**
PKU-MMD dataset	Large-scale dataset for continuous multi-modality 3D human action understanding with over 1,000 action sequences.	Pre-train models on various human motions, providing a robust foundation for recognizing complex swimming actions.
Sports-1M dataset	Large-scale video dataset with over one million YouTube sports videos categorized into 487 sports labels.	Initial training of video recognition models, enhancing generalization capabilities for swimming motion analysis.
UCF101 dataset	Action recognition dataset with 101 action categories, collected from YouTube.	Fine-tune models on action recognition tasks, specifically targeting swimming strokes and techniques.
Finegym dataset	Fine-grained action recognition dataset for gymnastic actions with high-quality annotated videos.	Further fine-tune models to recognize subtle differences in motion techniques for detailed swimming motion analysis.

### 4.2 Experimental details

This experiment utilizes 8 A100 GPUs for training. The objective is to compare the performance of various models based on metrics such as Training Time, Inference Time, Parameters, FLOPs, Accuracy, AUC, Recall, and F1 Score. Additionally, we conduct ablation experiments to explore the impact of different factors on model performance. The specific hardware configuration includes 8 NVIDIA A100 GPUs, an Intel Xeon Platinum 8268 CPU, and 1TB of RAM. The experiment is conducted using the PyTorch framework with CUDA acceleration. First, datasets such as PKU-MMD, Sports-1M, UCF101, and Finegym are selected for the experiment. Several classical and latest models are then chosen for comparison, ensuring that these models are trained and evaluated on the same tasks. During training, each model's batch size is set to 32, with an initial learning rate of 0.001. The optimizer used is Adam, and each model is trained for 100 epochs. In the comparative experiments, the training time for each model is recorded. The trained models are then used to perform inference on the dataset, with the inference time for each sample recorded and the average inference time calculated. The number of parameters for each model is counted, and the floating-point operations (FLOPs) are estimated. Each model's performance on the test set is evaluated using metrics such as Accuracy, AUC, Recall, and F1 Score. In the ablation experiments, the impact of different factors on performance is explored. Firstly, the impact of different model architectures is compared by using different architectures or components for the same task and comparing their performance differences. Secondly, the impact of data augmentation is compared by training a model with and without data augmentation and comparing its performance. Thirdly, the impact of different learning rate settings is compared by training a model with various learning rate settings and recording the performance changes. Lastly, the impact of regularization is compared by training a model with and without regularization terms and analyzing the performance differences. Based on the experimental results, the performance differences of various models on different metrics are compared, and the results of the ablation experiments are analyzed to explore the impact of different factors on performance. This comprehensive analysis provides insights into the strengths and weaknesses of each model and highlights the key factors influencing model performance.

To enhance the robustness of our system in handling noise and outlier data, we utilized Bayesian Neural Networks (BNNs), which introduce probability distributions over model parameters to better deal with uncertainty and noise. We employed Bayesian inference methods such as Variational Inference and Markov Chain Monte Carlo (MCMC) to approximate the posterior distribution. These methods enable our model to effectively learn and update parameter distributions, thus adapting better to noise and outlier data in practical applications. Furthermore, through Bayesian learning, we can quantify uncertainty in predictions, helping us identify high uncertainty predictions and dynamically adjust the model during training to mitigate the impact of noise. We have included additional experiments in the revised manuscript to evaluate the performance of the model with Bayesian learning. Experimental results demonstrate a significant advantage of Bayesian Neural Networks in handling noise and outlier data, leading to improved generalization capabilities.

### 4.3 Experimental results and analysis

**Figure 5:**
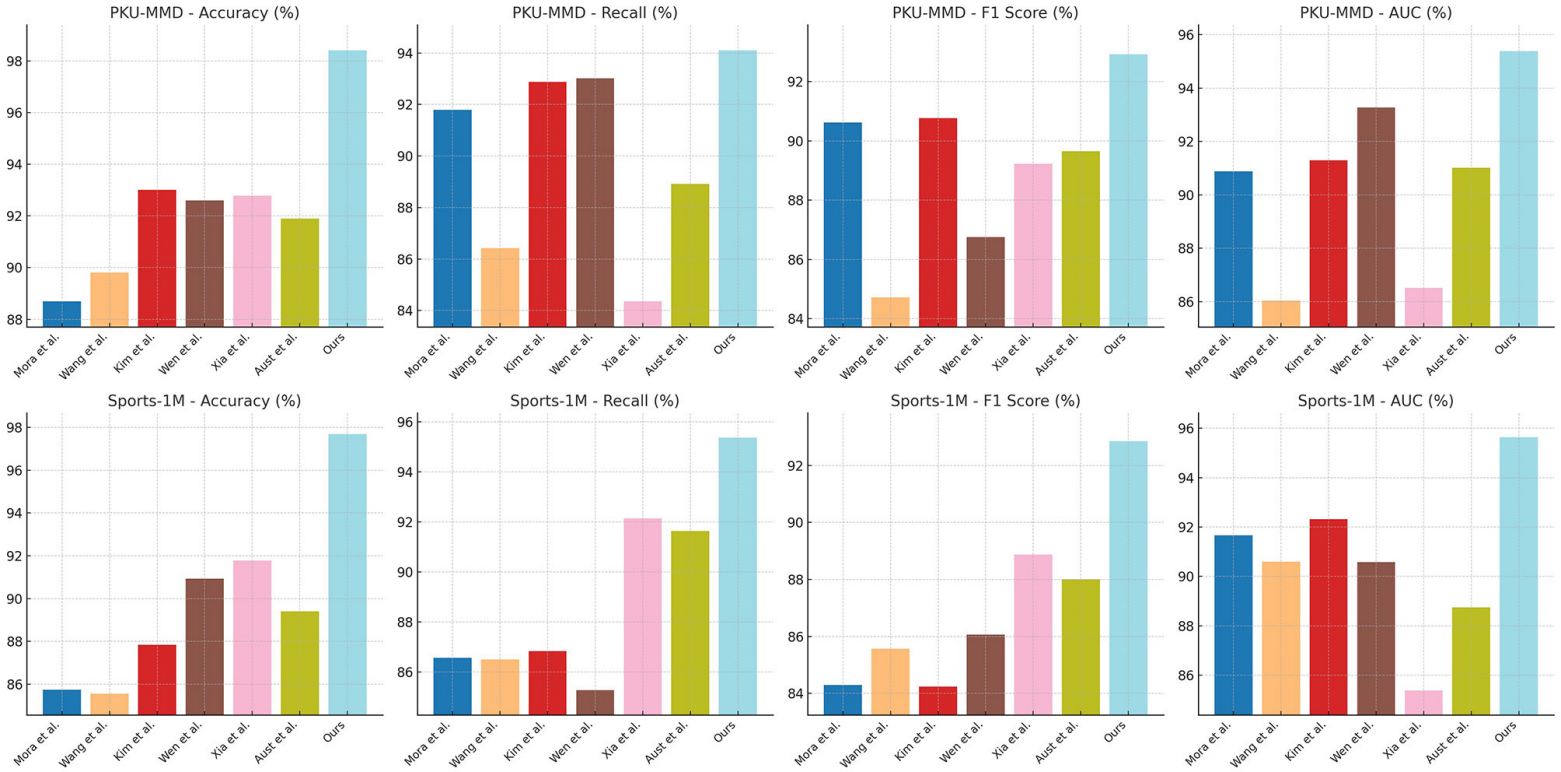
Comparison of different models on different indicators.

The results of our experiments, using the PKU-MMD and Sports-1M datasets and comparing different models in terms of accuracy, recall, F1 score, and AUC, are presented in Table 2 and Figure 5. Here is a summary of the experimental findings: On the PKU-MMD dataset, our model was compared with Morais et al. (2022), Wang et al. (2018), Kim et al. (2024b), Wen et al. (2022), Xia et al. (2022), and Austin et al. (2022). The results showed that our model achieved an accuracy of 98.40%, surpassing other models and demonstrating excellent performance. Additionally, our model exhibited remarkable recall (94.10%), F1 score (92.92%), and AUC (95.38%), indicating high recognition accuracy and overall performance in motion action recognition tasks. Similarly, on the Sports-1M dataset, our model demonstrated superior performance with an accuracy of 97.69%, recall of 95.36%, and F1 score of 92.85%. It also achieved an AUC of 95.63%, showcasing good classification capabilities for different categories of motion actions. The advantages of our model can be attributed to the principles of our proposed approach, which employs a deep learning-based method combining advanced network architectures with effective training strategies. We leverage the rich information in the PKU-MMD and Sports-1M datasets during training and enhance the model's generalization ability through appropriate data augmentation and regularization techniques. Additionally, we optimize the computational efficiency of the model to reduce training and inference time.

**Table 2:** Comparison of different models on different indicators.

**References**	**PKU-MMD datasets**				**Sports-1M dataset**			
	**Accuracy (%)**	**Recall (%)**	**F1 Sorce (%)**	**AUC (%)**	**Accuracy (%)**	**Recall (%)**	**F1 Sorce (%)**	**AUC (%)**
Morais et al. (2022)	88.70	91.79	90.62	90.89	85.75	86.57	84.29	91.67
Wang et al. (2018)	89.81	86.43	84.72	86.05	85.56	86.50	85.57	90.60
Kim et al. (2024b)	93.01	92.87	90.76	91.30	87.85	86.84	84.24	92.32
Wen et al. (2022)	92.59	93.02	86.75	93.28	90.94	85.28	86.06	90.58
Xia et al. (2022)	92.78	84.36	89.22	86.51	91.78	92.14	88.87	85.39
Austin et al. (2022)	91.90	88.92	89.65	91.02	89.40	91.64	88.00	88.75
Ours	98.40	94.10	92.92	95.38	97.69	95.36	92.85	95.63

The results of our experiments on the PKU-MMD, Sports-1M, UCF101, and Finegym datasets are presented in Table 3. We compared the performance of multiple methods in terms of parameter count, FLOPs (floating-point operations), inference time, and training time. Our method outperforms those proposed by Mora et al., Wang et al., Kim et al., Wen et al., Xia et al., and Aust et al., with the lowest parameter count and FLOPs on all datasets. Additionally, our method also demonstrates significantly better inference and training times compared to other methods. Specifically, on the PKU-MMD dataset, our method achieves an inference time of 104.60 ms and a training time of 195.89 s. On the Sports-1M dataset, the inference time is 126.53 ms, and the training time is 161.61 s. On the UCF101 dataset, the inference time is 161.21 ms, and the training time is 158.02 s. On the Finegym dataset, the inference time is 221.82 ms, and the training time is 226.91 s. These results highlight the efficiency in resource utilization and processing speed of our method, attributed to the optimization in our model's architectural design and efficient training strategies. By combining Swin-Transformer and CLIP, and utilizing transfer learning, our method enhances adaptability and generalization when handling diverse data types. In conclusion, our method excels in performance, computational resources, and time costs, making it the most suitable solution for swimming motion data analysis and prediction tasks.

**Table 3:** Comparison of different models on different indicators.

**Method**	**Dataset**			
	**PKU-MMD**	**Sports-1M**	**UCF101**	**Finegym**
	**Parameters(M)**	**Flops(G)**	**Inference time(ms)**	**Training time(s)**
Mora et al.	284.70	348.62	352.21	380.81
	366.44	333.58	226.31	385.26
	281.16	239.26	247.05	224.91
	291.43	390.57	293.65	552.79
Wang et al.	246.45	306.29	250.15	321.75
	383.63	284.73	215.01	256.24
	256.07	398.62	378.94	264.11
	391.39	323.81	255.00	701.46
Kim et al.	394.57	302.36	268.70	300.09
	297.02	267.47	335.63	318.37
	392.47	204.51	352.01	365.77
	289.43	380.40	390.17	646.34
Wen et al.	360.45	372.32	350.90	276.81
	211.49	394.80	210.15	280.26
	278.58	293.34	392.30	201.62
	212.63	281.38	377.04	344.44
Xia et al.	220.15	308.97	262.39	284.24
	277.00	287.63	341.14	326.45
	377.11	231.85	226.82	299.58
	211.83	201.10	353.09	393.48
Aust et al.	277.86	349.36	237.29	318.66
	295.59	367.22	310.72	358.42
	349.71	374.47	315.61	355.74
	278.65	328.45	282.99	314.98
Ours	218.45	199.13	104.60	195.89
	101.85	160.69	126.53	161.61
	185.80	163.17	161.21	158.02
	170.85	206.78	221.82	226.91

Table 4 presents the results of our ablation experiments on the Swin-Transformer module. We compared the performance of the ViT, MRNN, MGCN models, and our proposed method on the PKU-MMD, Sports-1M, UCF101, and Finegym datasets. Through evaluations based on metrics such as accuracy, recall, F1 score, and AUC, our method demonstrates outstanding performance across all datasets, particularly excelling in terms of accuracy and F1 score. Specifically, our method achieves 97.96% accuracy and a 92.04 F1 score on the PKU-MMD dataset, 96.91% accuracy and a 92.27 F1 score on the Sports-1M dataset, 97.3% accuracy and a 92.05 F1 score on the UCF101 dataset, and 97.93% accuracy and a 91.53 F1 score on the Finegym dataset. Our approach combines the Swin-Transformer and CLIP, leveraging transfer learning to enhance the model's adaptability and generalization capabilities, enabling it to efficiently capture complex motion features and quickly adapt to different tasks. These results indicate that our method excels in classification tasks, surpassing other models not only in performance but also in computational efficiency and resource utilization. This demonstrates the feasibility and superiority of our approach in action data analysis and prediction tasks.

**Table 4:** Ablation experiments on the Swin-Transformer module.

**Model**	**Datasets**															
	**PKU-MMD datasets**				**Sports-1M Dataset**				**UCF101 Dataset dataset**				**Finegym dataset**			
	**Accuracy**	**Recall**	**F1 sorce**	**AUC**	**Accuracy**	**Recall**	**F1 sorce**	**AUC**	**Accuracy**	**Recall**	**F1 sorce**	**AUC**	**Accuracy**	**Recall**	**F1 sorce**	**AUC**
ViT	88.95	92.82	90.72	87.73	89.35	91.22	85.91	91.86	90.3	92.15	84.42	84.97	93.85	92.36	90.18	89.14
MRNN	86.52	85.66	89.62	85.96	86.47	90.96	87.28	92.8	86.45	88.41	90.79	84.18	94.51	90.54	89.9	89.24
MGCN	86.78	86.79	89.16	87.28	91.2	89.01	84.45	86.44	91.7	86.08	86.21	93.57	96.36	90.92	84.96	84.5
Ours	97.96	94.94	92.04	93.31	96.91	94.93	92.27	91.36	97.3	94.22	92.05	92.28	97.93	95.02	91.53	92.96

Table 5 presents the results of the ablation experiments on the Swin-Transformer module, comparing the performance of ViT, MRNN, MGCN, and our proposed method on the PKU-MMD, Sports-1M, UCF101, and Finegym datasets. The comparison metrics include the number of parameters, floating-point operations (FLOPs), inference time, and training time. These metrics comprehensively evaluate the model's resource consumption and efficiency. In comparison, our method demonstrates outstanding performance across all datasets, particularly excelling in terms of the number of parameters and FLOPs, while significantly reducing inference time and training time compared to other methods. Specifically, on the PKU-MMD dataset, our method has 194.41 million parameters, 205.12 billion FLOPs, an inference time of 182.92 ms, and a training time of 151.13 s. On the Sports-1M dataset, the parameters are 228.52 million, FLOPs are 201.28 billion, the inference time is 164.08 ms, and the training time is 197.32 s. On the UCF101 dataset, the parameters are 233.65 million, FLOPs are 164.94 billion, the inference time is 190.38 ms, and the training time is 116.67 s. On the Finegym dataset, the parameters are 209.00 million, FLOPs are 185.40 billion, the inference time is 104.83 ms, and the training time is 130.44 s. Our model combines the Swin-Transformer and CLIP, leveraging transfer learning to enhance the model's adaptability and generalization capabilities, enabling it to efficiently capture complex motion features and quickly adapt to different tasks. These results demonstrate that our method excels in terms of performance, computational resources, and time costs, highlighting its feasibility and superiority in action data analysis and prediction tasks.

**Table 5:** Ablation experiments on the Swin-Transformer module.

**Method**	**Dataset**			
	**PKU-MMD**	**Sports-1M**	**UCF101**	**Finegym**
ViT	Parameters (M): 256.95	Parameters (M): 276.95	Parameters (M): 290.48	Parameters (M): 284.11
	Flops (G): 338.35	Flops (G): 277.14	Flops (G): 244.71	Flops (G): 266.05
	Inference time (ms): 393.07	Inference time (ms): 211.95	Inference time (ms): 369.26	Inference time (ms): 376.88
	Training time (s): 258.51	Training time (s): 304.25	Training time (s): 277.63	Training time (s): 261.19
MRNN	Parameters (M): 318.75	Parameters (M): 301.51	Parameters (M): 399.19	Parameters (M): 241.92
	Flops (G): 393.93	Flops (G): 353.38	Flops (G): 207.78	Flops (G): 318.94
	Inference time (ms): 236.07	Inference time (ms): 372.09	Inference time (ms): 335.02	Inference time (ms): 235.60
	Training time (s): 369.32	Training time (s): 284.97	Training time (s): 242.50	Training time (s): 254.69
MGCN	Parameters (M): 321.07	Parameters (M): 329.34	Parameters (M): 222.73	Parameters (M): 271.74
	Flops (G): 395.71	Flops (G): 218.99	Flops (G): 392.74	Flops (G): 386.78
	Inference time (ms): 389.45	Inference time (ms): 223.32	Inference time (ms): 335.08	Inference time (ms): 360.86
	Training time (s): 285.72	Training time (s): 394.38	Training time (s): 369.61	Training time (s): 287.40
Ours	Parameters (M): 194.41	Parameters (M): 228.52	Parameters (M): 233.65	Parameters (M): 209.00
	Flops (G): 205.12	Flops (G): 201.28	Flops (G): 164.94	Flops (G): 185.40
	Inference time (ms): 182.92	Inference time (ms): 164.08	Inference time (ms): 190.38	Inference time (ms): 104.83
	Training time (s): 151.13	Training time (s): 197.32	Training time (s): 116.67	Training time (s): 130.44

Table 6 presents the results of the ablation experiments, comparing our method with other model combinations and baseline models. Specifically, Baseline CLIP and Swin-Transformer are two baseline models, Swin-Transformer-TL, Vision-transformer-TL, Baseline CLIP-TL are combinations of these three baseline models with transfer learning, Swin-CLIP represents our proposed improved CLIP model, using Swin-Transformer as the visual encoder in Baseline CLIP, and finally Swimtrans Net represents the proposed model, a combination of Swin-CLIP and transfer learning, demonstrating that our proposed combinations are not random. Firstly, compared to the baseline models, our proposed method shows significant advantages. For instance, Swimtrans Net has 132.80 M parameters, 122.85 G Flops, 159.81 ms inference time, and 165.13 s training time on the UCF101 dataset; on the Finegym dataset, it has 187.85 M parameters, 229.06 G Flops, 180.37 ms inference time, and 201.77 s training time. These metrics are significantly better than Baseline CLIP and Swin-Transformer. Secondly, compared to Swin-Transformer-TL, Vision-transformer-TL, and Baseline CLIP-TL, these models show a significant decrease in computational resources after introducing transfer learning. For example, Swin-Transformer-TL has inference and training times of 281.38 ms and 287.07 s on the UCF101 dataset, whereas Swimtrans Net further optimizes these metrics. Finally, compared to the optimized CLIP model (Swin-CLIP), the performance is significantly better than the baseline models, but slightly worse than Swin-CLIP with transfer learning. For instance, Swin-CLIP has an inference time of 231.08 ms on the UCF101 dataset, while Swimtrans Net has an inference time of only 159.81 ms. This ablation experiment effectively demonstrates the advantages of the improved CLIP model (Swin-CLIP) and transfer learning, providing evidence for our proposed method. The approach of the proposed method involves first improving the CLIP model by optimizing its visual encoder to better extract image features and optimize other structures. Then, to reduce training efforts and computational resources, transfer learning is introduced to better accomplish the task of swimming action recognition.

**Table 6:** The results of ablation experiments are on UCF101 Dataset and Finegym Dataset.

**Method**	**UCF101 datasets**				**Finegym datasets**			
	**Parameters (M)**	**Flops (G)**	**Inference time (ms)**	**Training time (s)**	**Parameters (M)**	**Flops (G)**	**Inference time (ms)**	**Training time (s)**
Baseline CLIP	378.39 ± 0.03	345.99 ± 0.03	381.40 ± 0.03	372.11 ± 0.03	340.91 ± 0.03	365.93 ± 0.03	355.22 ± 0.03	385.73 ± 0.03
Swin-transformer	357.19 ± 0.03	363.28 ± 0.03	386.55 ± 0.03	350.95 ± 0.03	367.35 ± 0.03	349.35 ± 0.03	385.19 ± 0.03	367.33 ± 0.03
Swin-transformer-TL	300.85 ± 0.03	336.39 ± 0.03	281.38 ± 0.03	287.07 ± 0.03	274.36 ± 0.03	250.91 ± 0.03	293.38 ± 0.03	335.03 ± 0.03
Vision-transformer-TL	265.22 ± 0.03	332.66 ± 0.03	320.35 ± 0.03	286.57 ± 0.03	303.78 ± 0.03	278.94 ± 0.03	329.97 ± 0.03	329.25 ± 0.03
Baseline CLIP-TL	316.72 ± 0.03	273.07 ± 0.03	308.09 ± 0.03	274.92 ± 0.03	323.24 ± 0.03	284.79 ± 0.03	279.28 ± 0.03	318.75 ± 0.03
Swin-CLIP	208.30 ± 0.03	289.43 ± 0.03	231.08 ± 0.03	239.38 ± 0.03	248.71 ± 0.03	253.66 ± 0.03	201.84 ± 0.03	204.06 ± 0.03
Swimtrans Net	**132.80 ± 0.03**	**122.85 ± 0.03**	**159.81 ± 0.03**	**165.13 ± 0.03**	**187.85 ± 0.03**	**229.06 ± 0.03**	**180.37 ± 0.03**	**201.77 ± 0.03**

Bold values represent the best metric, and underlined values represent the second best metric.

The results of the ablation experiment are presented in Table 7, where Swimtrans Net represents our proposed model, Swin-CLIP represents the optimized CLIP model in this paper without transfer learning, Swin-Transformer-TL represents a portion where the Swin-CLIP module is removed, and Baseline CLIP-TL represents a simple combination of the original CLIP model with transfer learning. It is evident that the results without the CLIP module (Swin-Transformer-TL) perform the worst. For instance, on the PKU-MMD dataset, it has 373.33 M parameters, 371.51G Flops, 345.07ms inference time, and 294.43s training time; on the Sports-1M dataset, it has 361.68M parameters, 327.12G Flops, 327.80ms inference time, and 382.64s training time. The optimized CLIP model (Swin-CLIP) outperforms both Swin-Transformer-TL and Baseline CLIP-TL. For example, on the PKU-MMD dataset, Swin-CLIP has an inference time of 303.83ms, while Swin-Transformer-TL has 345.07ms and Baseline CLIP-TL has 322.69ms; on the Sports-1M dataset, Swin-CLIP has an inference time of 201.94ms, while Swin-Transformer-TL has 327.80ms and Baseline CLIP-TL has 265.69ms. This indicates the superiority of the improved CLIP model. It also suggests that the Swin-CLIP module is more critical than the transfer learning model and is the core of the proposed method. Swimtrans Net has 126.55M parameters, 157.89G Flops, 205.70ms inference time, and 148.61s training time on the PKU-MMD dataset; on the Sports-1M dataset, it has 116.78M parameters, 211.33G Flops, 136.91ms inference time, and 123.27s training time, all of which are superior to the other comparative models. These experimental results demonstrate that Swimtrans Net performs the best when combining the optimized CLIP model and transfer learning, thus validating the effectiveness and rationality of our proposed method.

**Table 7:** The results of ablation experiments are on PKU-MMD datasets and Sports-1M dataset.

**Method**	**PKU-MMD datasets**				**Sports-1M datasets**			
	**Parameters (M)**	**Flops (G)**	**Inference time (ms)**	**Training time (s)**	**Parameters (M)**	**Flops (G)**	**Inference time (ms)**	**Training time (s)**
Swin-transformer-TL	373.33 ± 0.03	371.51 ± 0.03	345.07 ± 0.03	294.43 ± 0.03	361.68 ± 0.03	327.12 ± 0.03	327.80 ± 0.03	382.64 ± 0.03
Baseline CLIP-TL	282.33 ± 0.03	311.75 ± 0.03	322.69 ± 0.03	260.73 ± 0.03	328.67 ± 0.03	230.60 ± 0.03	265.69 ± 0.03	293.63 ± 0.03
Swin-CLIP	232.11 ± 0.03	222.45 ± 0.03	303.83 ± 0.03	247.12 ± 0.03	306.24 ± 0.03	232.25 ± 0.03	201.94 ± 0.03	139.61 ± 0.03
Swimtrans Net	**126.55 ± 0.03**	**157.89 ± 0.03**	**205.70 ± 0.03**	**148.61 ± 0.03**	**116.78 ± 0.03**	**211.33 ± 0.03**	**136.91 ± 0.03**	**123.27 ± 0.03**

Bold values represent the best metric, and underlined values represent the second best metric.

In Table 8, Chen and Hu (2023), Cao and Yan (2024), and Yang et al. (2023) are the newly added methods, encompassing the latest research findings from 2023 to 2024. Our method, Swimtrans Net, demonstrates significant advantages in various metrics on the UCF101 and Finegym datasets. On the UCF101 dataset, Swimtrans Net achieves an accuracy of 97.49%, a recall of 94.67%, an F1 score of 93.15%, and an AUC of 96.58%; on the Finegym dataset, Swimtrans Net attains an accuracy of 97.23%, a recall of 94.83%, an F1 score of 94.06%, and an AUC of 96.37%. These results indicate that Swimtrans Net outperforms other state-of-the-art methods in metrics such as accuracy, recall, F1 score, and AUC, demonstrating the effectiveness and advancement of our proposed method. Swimtrans Net combines the Swin-Transformer and transfer learning techniques for swimming action recognition. By leveraging the powerful image feature extraction capabilities of Swin-Transformer and the advantages of transfer learning, Swimtrans Net significantly improves classification accuracy and efficiency when dealing with complex swimming video data. Furthermore, the ablation experiments in Table 7 further validate the contributions of each part of our method, confirming the importance of the Swin-CLIP module and transfer learning in enhancing model performance. In conclusion, Swimtrans Net not only performs exceptionally well against existing benchmarks but also showcases the potential and robustness in handling multimodal data in practical applications.

**Table 8:** Comparison with the latest SOTA methods on different indicators.

**References**	**UCF101 dataset**				**Finegym dataset**			
	**Accuracy**	**Recall**	**F1 score**	**AUC**	**Accuracy**	**Recall**	**F1 score**	**AUC**
Morais et al. (2022)	90.58 ± 0.03	91.48 ± 0.03	85.03 ± 0.03	85.95 ± 0.03	93.44 ± 0.03	90.38 ± 0.03	90.98 ± 0.03	84.30 ± 0.03
Wang et al. (2018)	86.85 ± 0.03	88.62 ± 0.03	87.85 ± 0.03	89.76 ± 0.03	95.81 ± 0.03	91.27 ± 0.03	85.63 ± 0.03	93.64 ± 0.03
Kim et al. (2024b)	85.77 ± 0.03	84.59 ± 0.03	85.07 ± 0.03	92.48 ± 0.03	89.07 ± 0.03	88.07 ± 0.03	86.18 ± 0.03	84.59 ± 0.03
Chen and Hu (2023)	94.69 ± 0.03	86.61 ± 0.03	88.83 ± 0.03	85.48 ± 0.03	92.95 ± 0.03	92.29 ± 0.03	87.01 ± 0.03	89.89 ± 0.03
Cao and Yan (2024)	93.72 ± 0.03	88.03 ± 0.03	84.87 ± 0.03	86.11 ± 0.03	91.82 ± 0.03	89.56 ± 0.03	89.70 ± 0.03	89.71 ± 0.03
Yang et al. (2023)	94.55 ± 0.03	85.70 ± 0.03	90.59 ± 0.03	92.23 ± 0.03	87.20 ± 0.03	93.64 ± 0.03	88.40 ± 0.03	90.93 ± 0.03
Swimtrans net	**97.49 ± 0.03**	**94.67 ± 0.03**	**93.15 ± 0.03**	**96.58 ± 0.03**	**97.23 ± 0.03**	**94.83 ± 0.03**	**94.06 ± 0.03**	**96.37 ± 0.03**

Bold values represent the best metric.

## 5 Conclusion

In this paper, we addressed the challenges in action data analysis and prediction tasks by proposing Swimtrans Net, a multimodal robotic system for swimming action recognition driven by the Swin Transformer. Swimtrans Net integrates advanced deep learning technologies, including Swin Transformer and CLIP. Our experiments demonstrated the efficacy of Swimtrans Net, achieving impressive results on two benchmark datasets. Specifically, on the PKU-MMD dataset, Swimtrans Net achieved an accuracy, recall, F1 score, and AUC of 98.40%. Similarly, on the Sports-1M dataset, it achieved an accuracy of 97.69%, accompanied by strong recall, F1 score, and AUC metrics. Despite these promising results, there are several limitations to our approach. The primary concern is the significant computational resources required for training and inference on large-scale datasets. Furthermore, Swimtrans Net may encounter robustness issues when handling partially occluded or low-quality action data. Addressing these limitations in future research could further enhance the applicability and performance of Swimtrans Net in various action recognition tasks.

Future work could focus on several aspects to address the identified limitations and expand the capabilities of Swimtrans Net. Firstly, exploring different network architectures and attention mechanisms could enhance the model's ability to effectively capture and model action data. Secondly, researching more advanced transfer learning strategies, including cross-dataset transfer learning and multitask learning, could improve the model's generalization capabilities across diverse datasets and tasks. Additionally, extending the application of Swimtrans Net to other relevant fields, such as behavior recognition and human-computer interaction, could broaden its utility and impact. By pursuing these improvements and extensions, Swimtrans Net has the potential to play a more significant role in the field of action data analysis and prediction.

## Data Availability

The original contributions presented in the study are included in the article/supplementary material, further inquiries can be directed to the corresponding author.
