# Erratum: Swimtrans Net: a multimodal robotic system for swimming action recognition driven via Swin-Transformer

**DOI:** 10.3389/fnbot.2024.1508032

**Published:** 2024-10-21

**Authors:** Frontiers Production Office

**Affiliations:** Frontiers Media SA, Lausanne, Switzerland

**Keywords:** Swin-Transformer, CLIP, multimodal robotic, swimming action recognition, transfer learning

Due to a production error, the email address of the corresponding author, Xiaoyu Yue, was mistakenly written as “yuexiayu@njtech.edu.cn”. The correct email address is “yuexiaoyu@njtech.edu.cn”. A correction has been made to the **Correspondence** section:

^*^ **Correspondence:**

Xiaoyu Yue

yuexiaoyu@njtech.edu.cn

The publisher apologizes for this mistake. The original version of this article has been updated.

